# Sexual Function in Chinese Women with Polycystic Ovary Syndrome and Correlation with Clinical and Biochemical Characteristics

**DOI:** 10.1007/s43032-021-00612-4

**Published:** 2021-06-02

**Authors:** Xuanxuan Tian, Xiangyan Ruan, Juan Du, Juan Wang, Dongmei Yin, Jiaojiao Cheng, Rui Ju, Alfred O. Mueck

**Affiliations:** 1 Department of Gynecological Endocrinology, Beijing Obstetrics and Gynecology Hospital, Capital Medical University, Beijing, China; 2 Research Centre for Women’s Health and University Women’s Hospital of Tuebingen, University of Tuebingen, Tuebingen, Germany

**Keywords:** Sexual function, Polycystic ovarian syndrome, Sexual dysfunction, Correlation

## Abstract

To investigate sexual function in Chinese women with polycystic ovary syndrome (PCOS) and to explore the correlation with clinical and biochemical characteristics. A cross-sectional study was designed in 1000 PCOS women, aged 18–45 years, via the Chinese version of Female Sexual Function Index (FSFI) evaluating sexual function, with additional questions possibly related to sexual life. Clinical and biochemical characteristics likely to affect sexual function were determined, including anthropometric indicators, serum levels of hormones, luteinizing hormone to follicle-stimulating hormone ratio (LH/FSH ratio), prolactin (PRL), total testosterone (TT), free androgen index (FAI), sex-hormone-binding globulin (SHBG), glucose, and lipid metabolism indicators. Nine hundred ten PCOS women participated in the study, 685 patients were included after screening, and 211 were suitable to detect correlations of clinical and biochemical characteristics with sex function parameters. The mean total FSFI score was 24.19 ± 2.8; 79.56% of the women were at risk of female sexual dysfunction (FSD). Women doing regular aerobic exercise and use of contraception had higher FSFI scores, while those with a desire to conceive and clinical signs of hyperandrogenism had lower FSFI scores. There were negative associations of FSFI scores with age and body fat distribution. No significant associations between FSFI scores and hormonal factors (surprisingly including SHBG) were found, except for total testosterone and satisfaction (OR = 0.976, *p* = 0.002). HOMA-IR was significantly related to reduced desire score (OR = 0.914, *p* = 0.004) and lubrication score (OR = 0.964, *p* = 0.044). PCOS was associated with a high risk of FSD (defined according to FSFI) in about 80% of the women in our study, and clinical characteristics play a more important role.

**Supplementary Information:**

The online version contains supplementary material available at 10.1007/s43032-021-00612-4.

## Introduction

Polycystic ovary syndrome (PCOS) is one of the most common endocrine disorders in women of reproductive age, with an estimated prevalence of 10% around the world [1] and 5.6% in Chinese women aged 19–45 years [2]. This complex endocrine disturbance reflects multiple variable biochemical and clinical manifestations such as hyperandrogenism (HA), insulin resistance, dyslipidemia, obesity, and frequently with menstrual irregularities, infertility, and increased risk of adverse pregnancy outcomes [3–5]. These signs and complications can also lead to psychosocial and emotional disturbances and seriously affect quality of life [6, 7].

Female sexual dysfunction (FSD) is a highly prevalent disorder worldwide, which includes disorders of desire, arousal, orgasm, and pain [8]. Prevalence of FSD may vary according to cultural, racial, and health status. In our center, we performed the first study in China to investigate 1010 young otherwise healthy women (i.e., no PCOS) via the Internet, whereby 60.2% were at risk of FSD [9]. Within another survey in Beijing, 63.3% had increased risk of FSD [10], whilst in Nanjing, the overall prevalence of FSD was 56.8% [11]. The status of FSD in Chinese women is therefore serious but unclear for PCOS patients up to now, although a lot of studies have been done on various populations and recently a meta-analysis is also published [12]. Current opinions on this topic in other countries are controversial. Some authors have reported impairment compared with the general population [13–17], but others did not find a significant reduction in sexual function for women with PCOS [18–20].

It already has been acknowledged that androgens can influence sexuality, especially in patients with hypoactive sexual desire disorder (HSDD), a severe form of sexual desire disorder [21–23]. Since PCOS often is associated with hyperandrogenemia, the question arises whether increased androgen levels of PCOS patients may have a beneficial effect on sexual function. Furthermore, some other biochemical and clinical markers also may affect sexual health. In view of multiple factors that can impair the sexual function of these patients, most research around the world focused on the main clinical characteristics related with FSD. Obesity, hirsutism, infertility, and low self-esteem have been described to have aversive effects on sexuality by causing body dissatisfaction and interfering with their feminine self-perception and marital problems. In fact, studies that go into the potentially biochemical factors are only sporadically reported [14, 24, 25] and their opinions were inconsistent. However, to our knowledge, no study related to sexual function among women with PCOS and investigating possible related clinical and biochemical characteristics has been published in China until now. The aim of the present study was therefore to evaluate this question.

## Materials and Methods

### Ethical Approval

The study was approved by the Ethics Committee and Institutional Review Board of Beijing Obstetrics and Gynecology Hospital, Capital Medical University, People’s Republic of China (protocol no. 2019-KY-039-01).

### Recruitment of the Study Population

This cross-sectional study was performed with 1000 consecutive women with PCOS aged 18–45 years, who attended the Department of Gynecological Endocrinology, Beijing Obstetrics and Gynecology Hospital, from 31 provinces of China during June 2019 to December 2019. All women coming during this defined recruitment time were included if they were eligible according to the inclusion and exclusion criteria and were also willing to answer sensitive questions about FSD. Informed consent was obtained from all patients. All the study data were kept confidential.

***Exclusion criteria*** were hyperprolactinemia, thyroid dysfunction, adrenal dysfunction, diabetes mellitus, pregnancy, previous history of ovarian surgery, and medication that could interfere with the function of the hypothalamic-pituitary-gonadal axis within 3 months before entering the study, such as using oral contraceptives. ***Inclusion criteria*** were sexual activity during the past 4 weeks, readiness to answer the questionnaires on sexual function and additional questions related to sociodemographic data, and other items with possible links to sexual life. Only patients with a diagnosis of PCOS based on the revised criteria of Rotterdam (2004) were included [26]. Polycystic ovary was diagnosed by the presence of 12 or more 2–9 mm follicles in each ovary and/or increased ovarian volume (> 10cm^3^). Oligo-anovulation was defined as oligomenorrhea (menstrual cycle of > 35 days) or amenorrhea (i.e., no menstrual bleeding during the last 3 months). Hyperandrogenism was clinically defined as the presence of clinical signs of hirsutism (modified Ferriman and Gallwey score > = 3), acne, and/or levels of total testosterone higher than 55.07 ng/dL (1.91 nmol/L). The severity of acne was determined by a grading system based on the number of lesions (including comedones and inflammatory lesions) and their spread on the face, back, and chest to classify it from mild to severe (mild, total lesion count < 20; moderate, total lesion count 20 < =to < 50; severe, total lesion count > = 50). The severity of hirsutism according to mFG score was also classified into 3 groups from mild to severe (none and mild, < 3; moderate, 3 < = to < 6; severe, > = 7).

### Assessment of Sexual Function

The Chinese version of FSFI was used to assess female sexual function [27], containing 19 questions that cover six domains: desire, arousal, lubrication, orgasm, satisfaction, and pain. The response options based on Likert-type scales were used to calculate separate domain scores and a total score. A total score of less than 26.55 was defined as FSD [28]. Individual subdomain scores were obtained by weighting as original score times severity score. Regarding cut-offs for the subdomains of the FSFI, Shindel et al. [29] suggested an arbitrary score of 33% of the maximum score in each domain. Both approaches were used throughout our study.

Further questions included whether participants had been sexually active within the last 4 weeks, use of contraception (including condoms, IUDs, and otherwise except for hormonal contraception), wish for conceive or fear of unwanted pregnancy, degree of clinical hyperandrogenism, frequency of aerobic exercise, and basic information like age, height, weight.

Because of the sensitivity of this issue for most Chinese women, every woman was first asked to evaluate themselves on the basis of questionnaires followed by a direct face to face interview with the doctor or medical staff to confirm correct understanding of the questions. We also used this study to inform our patients about the importance of a healthy sexual life and how to resolve possible problems.

### Assay Methods

Baseline blood samples (in early follicular phase and/or if in amenorrhea) were collected after overnight fasting for at least 12 h. The levels of FSH (follicle-stimulating hormone), luteinizing hormone (LH), prolactin (PRL), and total testosterone (TT) were measured using an Immulite 2000 (Diagnostic Products Corp, Los Angeles, CA) by the Reproductive Endocrine Laboratory at Beijing Obstetrics and Gynecology Hospital. Sexual hormone binding globulin (SHBG) was measured by a non-competitive liquid phase chemiluminescent immunoassay (Immulite 1000, EURO/Diagnostics Products Corp. Ltd. Los Angeles, CA). The intra-assay and inter-assay coefficients of variation were 4.1 and 5.8%, respectively. Biochemical parameters including fasting blood sugar (FBS), fasting insulin (FINS), triglycerides (TG), total cholesterol (TC), and low/high density lipoprotein cholesterol (LDL-C/HDL-C) were measured using a Chemistry Analyzer (Beckman Coulter LX20, USA) in the clinical chemistry laboratory. The homeostasis model of assessment insulin resistance (HOMA-IR) was calculated according to the suggested formula: [glucose (nmol/L) × insulin (μU/mL)/22.5]. Free androgen index (FAI) values were calculated as T (nmol/l) × 100/SHBG (nmol/l). Body fat distribution and percentage of fat were detected using a muscle function analyzer (MC/MES00. 042, Beijing) after overnight fasting for at least 12 h.

### Patient Sample Calculation

This is an observational cross-sectional study. Based on a single population proportion formula, minimum sample formula was calculated: N = u_α_^2^ × prevalence (1 − prevalence)/δ^2^. The prevalence of FSD was estimated as 60.2% according to our earlier Internet-based study in young Chinese women [9]; the evaluated error δ was 5%; the minimum sample content was *n* = (1.96^2^ × 0.602× 0.398)/0.05^2^ = 368. For our study, we were primarily able to recruit almost three times the number of patients during our defined recruitment time of 6 months (intent to treat analysis) and almost twice the number of patients who could finally be analyzed according to the study protocol (per protocol analysis).

### Statistics

Statistical evaluations were performed as per protocol analysis, i.e., with patients who met all the study protocol criteria. Excel 2010 software was used to establish and manage the database, and the Statistical Package for Social Sciences 17.0 for Windows (SPSS) was used for statistical analysis. Continuous variables were verified for normality by the Kolmogorov-Smirnov test. Values were described as mean ± standard deviation (SD) or median (interquartile range) based on normal distribution test and percent for categorical variables. The independent sample *t* test and one-way analysis of variance (ANOVA) were used to compare female sexual function scores. Spearman’s correlation coefficients were obtained to find the association between total and domain scores and some biochemical and anthropometric variables. Multivariate linear regression analysis was used to estimate various risk factors for FSD and to control for confounding factors. Two-sided *p* values < 0.05 were considered to be significant.

## Results

### General Characteristics

A total of 910 PCOS women participated in the study (91.0%, intent to treat study group), and 685 women (68.5%, per protocol study group) completed all questions of the FSFI and answered further questions regarding basic characteristics and information with possible links to sexual life. The considerable lower number of women in the protocol group must be seen in context with the fact that China is a conservative nation and a lack of sex education is one of its characteristics. Until recently, few Chinese women were willing to discuss sexual problems. The mean age of participants was 29.02 ± 4.17 years; interquartile range was 26–32 years (50.0%, *n* = 343), with the youngest being 20 and the oldest 41. The mean BMI was 24.48 ± 4.47 kg/m^2^, and 35.62% of participants had BMI > = 25 kg/m^2^. Four hundred fifty-eight (66.86%) participants wished for a child, 220 (32.12%) used of contraception, and 34 (4.96%) fear of unwanted pregnancy currently. Women with severe hirsutism and/or acne accounted for 85 (12.41%), moderate for 262 (38.25%), and none and mild for 338 (49.34%) participants. Three hundred sixty-two (52.85%) participants had < 1 time of aerobic exercise per week, 294 (42.92%) had about once, and 29 (4.23%) had > = 2 times of aerobic exercise per week. The general characteristics of the study population are given in Table 1.

**Table 1:** Anthropometric and clinical characteristics of Chinese women with PCOS (*n* = 685)

Variable	PCOS	Percentage
Age (y), mean (SD)	29.02(4.17)	
20–25, *n* (%)	76	11.09
25–35, *n* (%)	542	79.12
35–45, *n* (%)	67	9.78
BMI (kg/m^2^), mean (SD)	24.48 (4.47)	
< 25 kg/m^2^, *n* (%)	441	64.38
> = 25 kg/m^2^, *n* (%)	244	35.62
Contraception		
Yes, *n* (%)	220	32.12
No, *n* (%)	465	67.88
Wish for conceive		
Yes, *n* (%)	458	66.86
No, *n* (%)	227	33.14
Fear of unwanted pregnancy		
Yes, *n* (%)	34	4.96
No, *n* (%)	651	95.04
Hirsutism and/or acne		
None and mild, *n* (%)	338	49.34
Moderate, *n* (%)	262	38.25
Severe, *n* (%)	85	12.41
Aerobic exercise (times per week)		
< 1, *n* (%)	362	52.85
1–2, *n* (%)	294	42.92
> = 2, *n* (%)	29	4.23

*BMI* body mass index

### Biochemical Characteristics

Data from a total of 211 patients were adequate after screening to perform further correlation analyses. These biochemical characteristics are shown in Table 2. The mean body fat distribution and body fat percentage of participants were 209.30 ± 55.19 g/cm and 43.06 ± 5.75%, respectively. The mean levels of PRL, SHBG, and lipid metabolism indicators (TG, TC, HDL-C, and HDL-C) were 12.25 ± 5.78 ng/ml; 41.34 ± 24.09 nmol/l; and 2.00 ± 1.09, 4.52 ± 0.92, 1.25 ± 0.36, and 2.78 ± 0.71 mmol/L, respectively. The levels of TT, FBS, FINS, LH to FSH ratio, FAI, and HOMA-IR were non-normally distributed, described as median (interquartile range) as follows, 46.76 (18.46) ng/dl, 4.90 (0.51) mmol/L, 11.40 (7.04) uIU/ml, 2.37 (0.91), 4.12(5.34), and 2.51 (1.58), respectively.

**Table 2:** Biochemical characteristics of Chinese women with PCOS (*n* = 211)

Variable	PCOS
Body fat distribution (g/cm), mean (SD)	209.30 (55.19)
Body fat percentage (%), mean (SD)	43.06 (5.75)
LH to FSH ratio, median (IQR)	2.37 (0.91)
PRL (ng/ml), mean (SD)	12.25 (5.78)
TT (ng/dl), median (IQR)	46.76 (18.46)
SHBG (nmol/l), mean (SD)	41.34 (24.09)
FAI, median (IQR)	4.12(5.34)
FBS (mmol/L), median (IQR)	4.90 (0.51)
FINS (uIU/ml), median (IQR)	11.40 (7.04)
HOMA-IR, median (IQR)	2.51 (1.58)
TG (mmol/L), mean (SD)	2.00 (1.09)
TC (mmol/L), mean (SD)	4.52 (0.92)
HDL-C (mmol/L), mean (SD)	1.25 (0.36)
LDL-C (mmol/L), mean (SD)	2.78 (0.71)

*LH* luteinizing hormone, *FSH* follicle-stimulating hormone, *PRL* prolactin, *TT* total testosterone, *SHBG* sex hormone-binding globulin, *FAI* free androgen index, *FBS* fasting blood sugar, *FINS* fasting insulin, *HOMA-IR* homeostasis model assessment for insulin resistance index, *TG* triglycerides, *TC* total cholesterol, *HDL-C* high-density lipoprotein cholesterol, *LDL-C* low-density lipoprotein cholesterol, HOMA-IR was calculated using following formula: [glucose (nmol/L) × insulin (μU/mL)/22.5]

### Sexual Dysfunction Parameters

The total FSFI scores and subscores are shown in Table 3. For the total FSFI score, a cut-off value of 26.55 was chosen, score of 33% of the maximum score in each domain, a cut-off value of 1.98 for each domain, respectively. Lower scores are related to a higher likelihood of sexual problems. Regarding the total FSFI score (mean score 24.19 ± 2.84), 545 participants were considered to be at high risk of sexual dysfunction (79.56%). Regarding the subscores, 66 were considered at high risk of dysfunction for lubrication (9.64%), 38 for orgasm (5.55%), 18 for desire (2.63%), 16 for pain (2.34%), 11 for arousal (1.61%), and 3 for satisfaction (0.44%).

**Table 3:** FSFI scores and proportion of participants with FSFI score < 26.55 (*n* = 685)

Score	Mean	SD	Maximum	Minimum	Range	Number at risk of sexual dysfunction	Percentage at risk of sexual dysfunction (%)
Total FSFI	24.19	2.84	32.5	10.4	22.1	545	79.56
Desire	3.58	0.82	6.0	1.2	4.8	18	2.63
Arousal	3.94	0.76	6.0	1.2	4.8	11	1.61
Lubrication	4.48	0.62	6.0	1.8	4.2	66	9.64
Orgasm	3.83	0.98	6.0	1.2	4.8	38	5.55
Satisfaction	4.32	0.78	6.0	1.2	4.8	3	0.44
Pain	4.04	0.85	6.0	1.2	4.8	16	2.34

*FSFI* female sexual function index.

### Comparison of FSFI Scores for General Characteristics

**Fig. 1:**
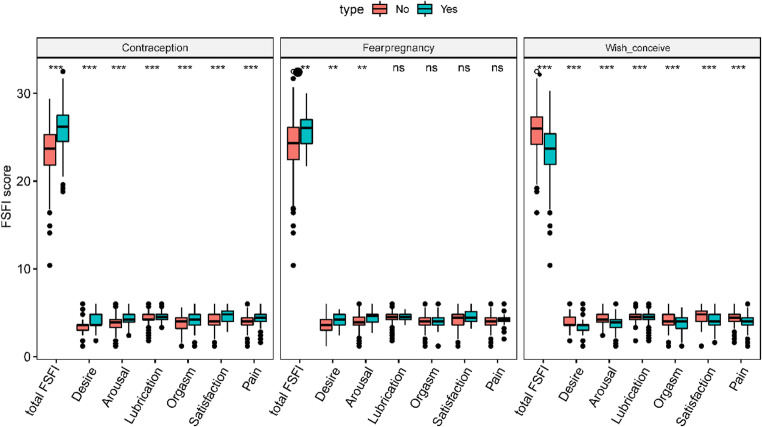
Comparison of mean FSFI scores for contraception, fear-pregnancy and wish-conceive. *** *p* < 0.001; ** *p* < 0.01; ns: there was no significant difference

Using the *t* test, a comparison of the mean scores showed that women who used no form of contraception, compared with contraceptive users, had lower total FSFI scores (*p* < 0.001) and also lower scores for desire, arousal, lubrication, orgasm, satisfaction, and pain (all *p* < 0.001). Women who wished for conceive had lower scores of total FSFI and lower scores of all domains (all *p* < 0.001). Women who feared an unwanted pregnancy had higher scores of total FSFI and also higher scores for the domains of desire and arousal (all *p* < 0.01) (Fig. 1).

**Fig. 2:**
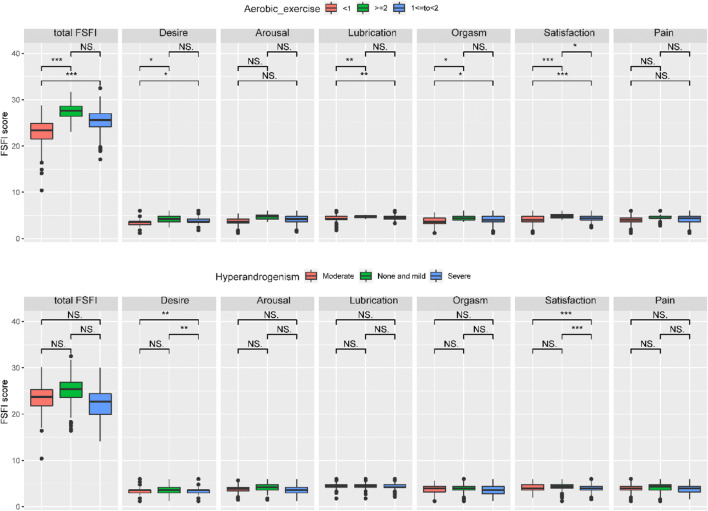
Comparison of mean FSFI scores for aerobic exercise and hyperandrogenism. *** *p* < 0.001; ** *p* < 0.01, *p < 0.05; NS: there was no significant difference

Using ANOVA, we found that women performing regular aerobic exercise (> = 1 time per week) had higher total FSFI scores compared with women who rarely exercised (*p* < 0.001). The same was true for desire (*p* < 0.05), lubrication (*p* < 0.01), orgasm (*p* < 0.05), and also satisfaction (*p* < 0.001) scores (group 1, < 1 time per week; group 2, 1 < = to < 2 times per week; group 3, > = 2 times per week). Women in group 3 had higher satisfaction score than that in group 2. However, there were no significant differences comparing the group 3 to group 2 in total score and other domains. Women with severe clinical signs of hyperandrogenism showed lower desire (*p* < 0.01) and satisfaction (*p* < 0.001) scores than that of none to mild and moderate groups and no difference between the latter two groups (Fig. 2).

### Spearman’s Correlations Analysis

The details of Spearman’s correlations of sex function and its domains with the clinical and biochemical features are shown in Table 4. There were significant negative correlations between age, BMI, body fat percentage, body fat distribution, and FSFI scores, and there were no significant associations between FSFI scores and hormonal parameters, except for total testosterone in relation to satisfaction (*r* = −0.139, *p* = 0.044) and FAI associated with increased orgasm score (*r* = 0.132, *p* = 0.046).

**Table 4:** Correlations of Female Sexual Function Index and its domains with the clinical and biochemical features of women with PCOS (*n* = 211)

Clinical and biochemical characteristics	Total FSFI		Desire		Arousal		Lubrication		Orgasm		Satisfaction		Pain	
	r	P	r	P	r	P	r	P	r	P	r	P	r	P
Age (y)	−0.567	< 0.001^**^	−0.299	< 0.001^**^	−0.305	< 0.001^**^	−0.294	< 0.001^**^	−0.395	< 0.001^**^	−0.448	< 0.001^**^	−0.197	< 0.001^**^
BMI (kg/m^2^)	−0.200	< 0.001^**^	−0.165	< 0.001^**^	−0.148	< 0.001^**^	−0.106	0.005^**^	−0.048	0.110	−0.139	< 0.001^**^	0.020	0.608
Body fat distribution (g/cm)	−0.103	0.137	−0.096	0.164	−0.198	0.004^**^	−0.086	0.214	0.005	0.944	0.008	0.905	0.017	0.807
Body fat percentage (%)	−0.169	0.014^*^	−0.109	0.113	−0.193	0.005^**^	−0.163	0.018^*^	−0.033	0.637	−0.115	0.096	0.065	0.346
LH to FSH ratio	0.113	0.103	0.063	0.365	−0.059	0.392	0.032	0.647	0.066	0.341	0.081	0.240	0.073	0.288
PRL (ng/ml)	−0.101	0.145	−0.024	0.733	0.020	0.775	0.061	0.380	−0.125	0.070	−0.074	0.284	−0.027	0.693
TT (ng/dl)	0.007	0.924	−0.016	0.817	−0.084	0.225	0.125	0.069	−0.049	0.481	−0.139	0.044^*^	0.092	0.185
SHBG (nmol/l)	0.011	0.870	0.004	0.958	−0.068	0.324	−0.054	0.431	0.127	0.066	−0.034	0.624	−0.018	0.793
FAI	−0.038	0.579	−0.080	0.250	0.007	0.922	0.101	0.142	0.132	0.046^*^	0.042	0.541	0.059	0.392
FBS (mmol/L),	0.005	0.938	−0.005	0.944	−0.037	0.590	0.085	0.219	0.043	0.536	−0.068	0.324	−0.016	0.815
FINS (uIU/ml)	0.108	0.118	0.071	0.302	0.023	0.737	0.084	0.222	0.123	0.076	0.050	0.474	0.074	0.283
HOMA-IR	0.080	0.248	−0.152	0.028^*^	−0.001	0.948	−0.189	0.006^**^	0.120	0.081	0.090	0.195	−0.046	0.510
TG (mmol/L)	0.077	0.264	−0.029	0.677	−0.024	0.732	−0.025	0.719	0.085	0.220	0.038	0.586	−0.046	0.503
TC (mmol/L)	−0.006	0.933	0.026	0.705	−0.006	0.935	0.063	0.366	0.058	0.401	−0.065	0.345	−0.048	0.492
HDL-C (mmol/L)	0.142	0.040^*^	−0.047	0.493	0.015	0.832	−0.101	0.145	−0.022	0.752	−0.035	0.612	−0.047	0.495
LDL -C (mmol/L)	−0.100	0.146	−0.019	0.788	−0.057	0.408	−0.014	0.838	−0.060	0.383	−0.116	0.094	−0.039	0.576

*BMI* body mass index, *LH* luteinizing hormone, *FSH* follicle-stimulating hormone, *PRL* prolactin, *TT* total testosterone, *SHBG* sex hormone-binding globulin, *FAI* Free androgen index, *FBS* fasting blood sugar, *FINS* fasting insulin, *HOMA-IR* homeostasis model assessment for insulin resistance index, *TG* triglycerides, *TC* total cholesterol, *HDL-C* high-density lipoprotein cholesterol, *LDL-C* low-density lipoprotein cholesterol

*p < 0.05. **p < 0.01

HOMA-IR was significantly related to reduced desire score (*r* = −0.152, *p* = 0.028) and lubrication score (*r* = −0.189, *p* = 0.006). We observed a positive significant correlation between the total FSFI score and HDL-C level (*r* = 0.142; *p* = 0.04). There were no significant associations between sexual function scores and serum TG, TC, and LDL-C levels.

### Multivariate Linear Regression Analysis

**Fig. 3:**
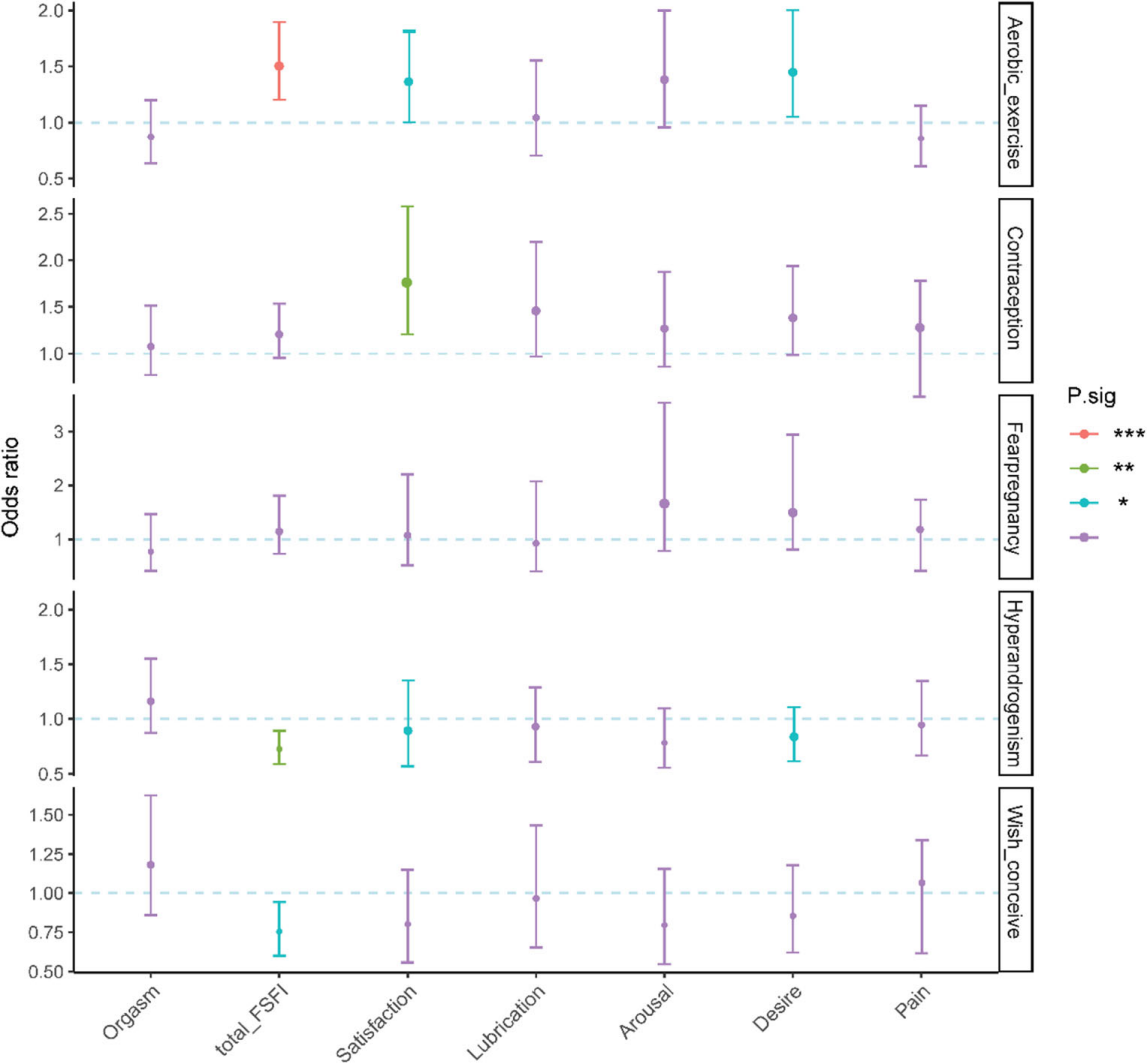
Multivariate generalized linear regression analysis of FSFI scores with general characteristics. *** *p* < 0.001; ** *p* < 0.01; *p < 0.05

According to the odds ratio (OR) and *p* value, aerobic exercise and use of contraception were protective factors, while hirsutism and/or acne and wish for conceive were remaining significant risk factors for FSD (Fig. 3). Aerobic exercise was positively correlated with total FSFI (OR = 1.503, *p* < 0.001), desire (OR = 1.412, *p* = 0.047), and satisfaction score (OR = 1.399, *p* = 0.037). The use of contraception was positively correlated with satisfaction (OR = 1.689, *p* = 0.003). Hirsutism and/or acne was negatively correlated with total FSFI (OR = 0.791, *p* = 0.008), desire (OR = 0.725, *p* = 0.018), and satisfaction score (OR = 0.905, *p* = 0.031). Wish for conceive was negatively correlated with total FSFI (OR = 0.749, p = 0.032).

**Fig. 4:**
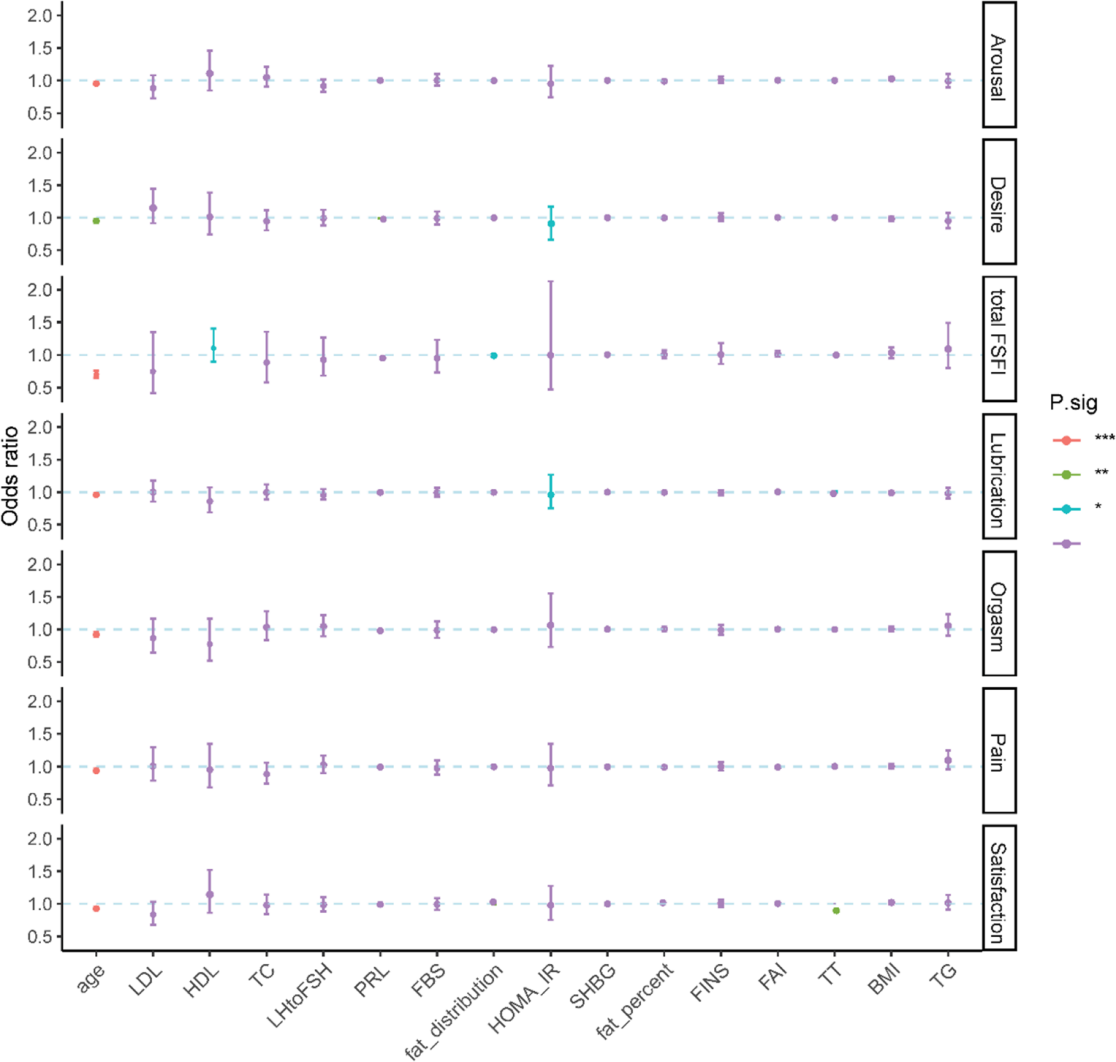
Multivariate linear regression analysis of FSFI scores with clinical and biochemical characteristics. *** *p* < 0.001; ** *p* < 0.01; *p < 0.05

The details of multivariate linear regression analysis of FSFI scores with clinical and biochemical characteristics are shown in Fig. 4. Age was a significant risk factor for FSD, negatively correlated with total FSFI score and for all domains (all *p* < 0.001). Fat distribution was negatively correlated with total FSFI score (OR = 0.993, *p* = 0.042), HOMA-IR was negatively correlated with desire (OR = 0.914, *p* = 0.004) and lubrication (OR = 0.964, *p* = 0.044) score, and TT was negatively correlated with satisfaction score (OR = 0.976, *p* = 0.002). Meanwhile, HDL-C seemed to be a protective factor for FSD, positively correlated with total FSFI score (OR = 1.119, *p* = 0.015).

## Discussion

This survey estimates the prevalence of FSD of 685 patients with PCOS coming from 31 provinces of China, i.e., mostly from all over China. As the first official Obstetrics and Gynecology Hospital in “New China,” our “Department of Gynecological Endocrinology” cares for the largest number of PCOS patients in China. Nevertheless, we experienced some recruitment difficulties, because one of the main inclusion criteria was that women were asked to answer voluntarily, but completely and correctly, questions regarding their sexual life to obtain scores of FSFI. Until recently, only few Chinese women were willing to discuss sexual problems. Therefore, many primarily eligible women were not willing to participate in this study, which may have led to a selection bias of participants (see “limitations” of our study). However, despite this recruitment problem, to our knowledge, this is the first study using large sample data to investigate the current sex life of women with PCOS in China.

About 80% of our patients were at high risk of FSD, higher than in the general population in China [9–11], and also higher than reported in some other studies in the USA, Iran, and Malaysia, with prevalence ranges between 27.2 and 62.5% [14–16]. The higher prevalence of FSD in Chinese PCOS patients may be due in part to different demographic characteristics of the women and their social and cultural background, such as the widespread lack of sexual knowledge, conservative beliefs, and neglect of FSD in China. Among the six domains of FSD, lubrication was found to be the most compromised sexual domain in our study, followed by disorders of orgasm and desire. Our earlier Internet-based study in 1010 women found that the top three dysfunctions were pain, orgasm, and desire [9]. Besides the fact that these women did not have PCOS, the reason for the difference may be that the group of the respondents to our Internet-based questionnaires were younger than the patients in our present face to face study.

Regarding our analysis to find associations with clinical characteristics and sexual function parameters, we found a significantly higher risk of FSD in our largest group of patients, who were women who attended because of *pressure of fertility problems.* This is in line with several other studies, where psychosexual implications were found to cause profound emotional distress which further could aggravate infertility problems [30–32]. In contrast, in our study *contraceptive users* had increased sexual function with higher satisfaction scores than those who used no form of contraception. This is consistent with our earlier Internet-based study [9]. Controversial results can be found in other studies; however, these did not investigate PCOS patients [33, 34].

We found women performing *regular exercise* had higher scores in total, desire, and satisfaction scores compared with women who rarely exercised, as confirmed in other studies in recent years. These studies found aerobic exercise improved sexual function and reduced the anxiety and depression of women with PCOS [35, 36]. Furthermore, aerobic exercise interferes in their cognitive-emotional dimension of body image, thus affecting sexual function [36].

*Clinical signs of hyperandrogenism* (hirsutism and/or acne) are significantly associated with a higher risk of FSD [18, 37]. Likewise, our study found that these women had lower desire and satisfaction scores. In fact, many PCOS patients with clinical hyperandrogenism have increased androgen levels [38]. The real mechanism of androgen on sexual desire is unknown. With the context of some patients with hypoactive sexual desire disorder (HSDD) can be treated with testosterone, an explanation for this obvious paradox may be that the negative appearance due to clinical hyperandrogenism (such as severe acne, hirsutism, alopecia) is one of the most important psychological factors behind FSD [39–41]. These negative effects for patients with PCOS may not be compensated for by the possibly beneficial effects of increased androgen levels. Another explanation could be that some studies showed significant positive correlations between psycho-emotional disorders such as depression and increased serum androgens, although a causal relationship is unclear [42–44]. There are therefore still unanswered questions regarding the effects of androgens in women with FSD, especially for PCOS women.

From other factors evaluated in our study, a clear association was found for *obesity* with obviously decreased sexual function. It already is known that obesity and sexual function are associated through various mental and physical pathways. Obesity increases the risk of cardiometabolic diseases that are also associated with lower sexual function, such as type 2 diabetes, frequently seen in patients with PCOS [45], and increases the risk of endocrine hormone disorders associated with FSD [46]. Furthermore, anxiety and depression are more prevalent in the obese population and are linked to FSD [47, 48]. Thus obesity, which is very frequent in women with PCOS, may be a common etiological factor for decreased sexual function [49, 50]. As a consequence, in our clinical practice, we try to treat obesity, especially in women with PCOS who wish for conceive, asking the women to undergo a standardized diet and exercise program and possible also prescribing drugs for the treatment of obesity [51, 52].

Regarding *biochemical markers*, we found no significant relationship to the FSFI scores, except for total testosterone and decreased satisfaction (OR = 0.976, *p* = 0.002). The reason may be that PCOS women with elevated total testosterone levels tend to have a negative self-image, higher BMI, and various psycho-emotional disorders [24]. It should be mentioned that we did not find any significant association between SHBG and FSD, although higher SHBG decreases free testosterone which could induce changes in the sexual function scores. Given that testosterone binding to SHBG is a complex, multistep process that involves inter-binding site allostery [53], the roles of SHBG in regulating circulating testosterone concentrations have been debated extensively, and the value of SHBG as biochemical marker for the diagnosis of PCOS is critically discussed. The significance of changes in levels of SHBG and the free testosterone fraction needs further experimental research [54].

Regarding *glucose and lipid metabolic indicators*, HOMA-IR was significantly related to reduced desire score and lubrication score. Insulin resistance is the most common metabolic disorder in women with PCOS and is at the center of its pathophysiology, causes diabetes with direct or indirect relationships with dyslipidemia and metabolic syndrome, and is the topic of various studies including our own research [55, 56]. It’s mechanisms leading to sexual dysfunction include hyperglycemia, infections, vascular and neurological damage, and hormonal disorders [57]. Vascular abnormalities, including atherosclerotic damage and endothelial dysfunction, may be responsible for reducing the engorgement of the clitoris and for reducing lubrication of the vagina, leading to decreased arousal and dyspareunia during sexual intercourse [58]. Diabetic neuropathy may further contribute to the pathogenesis of sexual dysfunctions by altering both the normal transduction of sexual stimuli and the triggered sexual response [59, 60].

In addition, we observed a positive significant correlation between total FSFI score and HDL-C level, therefore HDL-C may be a beneficial factor for increasing sexual function. To our knowledge, this has not been suggested in other studies until now, and it seems worthy of being investigated within further research.

### Strengths and Limitations of the Study

The ***strengths*** of our study are that we report, to our knowledge, for the first time the different prevalences of FSD and the different scores of FSFI and its domains for a large population of Chinese patients with PCOS. It also is a strength that all women were patients from one center where data were collected and all clinical examinations were performed, the first officially acknowledged “Department of Gynecological Endocrinology” in China and the center with the longest and greatest experience in treating PCOS. Because the women came from all over the country, our results should be representative for China. We evaluated possible sexual problems using the well-validated FSFI in the form of a direct face to face interview, which is superior to surveys only made by phone or letters.

The main ***limitation*** is that our study is observational and cross-sectional. Thus, only associations can be described, no causal relationships. Another limitation may be selection bias: Due to the sensitive issue of sexual problems, women with higher education may have more often agreed to participate in this study, and another selection bias could be that patients with more severe symptoms travelled a long way to one specialized center.

PCOS patients are a very heterogeneous group regarding clinical features and hormonal parameters. It would be desirable to repeat our analysis within an even larger population to obtain sufficient patient samples in subgroups of PCOS, for example, stratified according to the four main phenotypes of PCOS to minimize variability and confounding factors. In addition, the status of mental health of PCOS women also is an important factor of sexual function, which needs specialized research. Further studies are needed to confirm our results, especially a case-control study including also data of distress, confidence, and self-esteem, because there are contrary arguments on the negative effect of PCOS. To determine causal relationships to FSD, further exploration involving intervention at regular healthcare visits is needed. Despite these limitations, we think that our study is a very step in this research, has discovered the seriousness of FSD in a large PCOS group in China, and could help to recognize and take this problem seriously in the long-term management of PCOS.

## Conclusion

The prevalence of women with PCOS at high risk of FSD in this study is higher than that in the general population in China and higher than in some other countries. We found various factors which could influence sexual function such as age, frequency of exercise, and wanting to become pregnant. Although several biochemical disturbances can influence the sexual function of patients with PCOS, our results highlight the role of typical signs of PCOS such as obesity, infertility, and clinical signs of hyperandrogenism. Hence, clinicians should regularly assess the clinical dimensions of PCOS as well as biochemical factors in terms of possible effects on sex life.

## Supplementary Information

ESM 1 (PDF 39 kb)
