# Supplementary figures and images for: Sexual Function in Chinese Women with Polycystic Ovary Syndrome and Correlation with Clinical and Biochemical Characteristics
Source: Reprod Sci. 2021 Jun 2;28(11):3181–92. doi: 10.1007/s43032-021-00612-4 (PMC8526448; doi:10.1007/s43032-021-00612-4)

# Univariate generalized linear regression

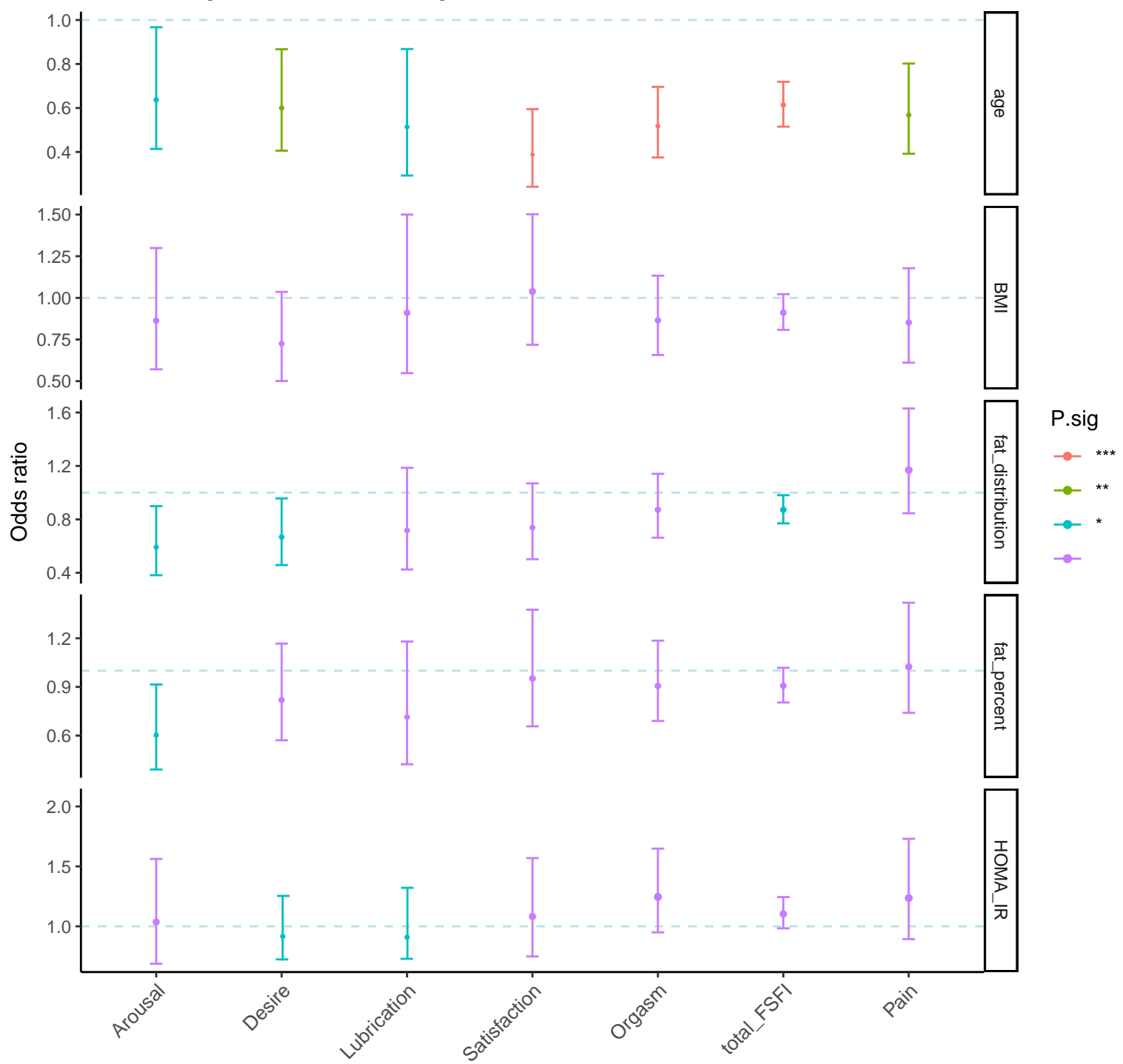

# Multivariate generalized linear regression

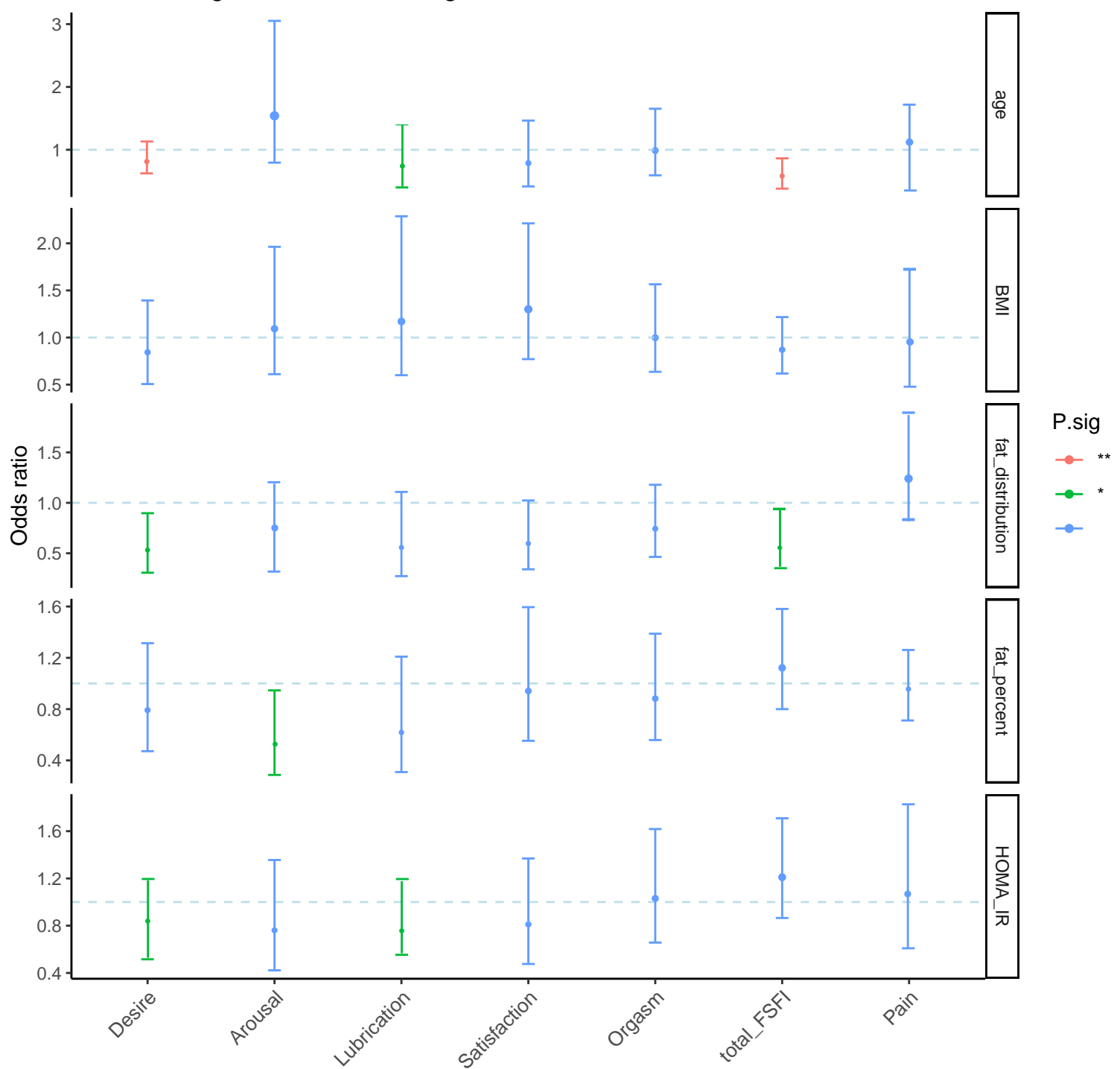

Supplement: Supplementary file 1 — (PDF 39 kb) [file 43032_2021_612_MOESM1_ESM.pdf]
